# Determination of myrislignan levels in BALB/c mouse plasma by LC-MS/MS and a comparison of its pharmacokinetics after oral and intraperitoneal administration

**DOI:** 10.1186/s12917-021-02990-y

**Published:** 2021-08-16

**Authors:** Jili Zhang, Hongfei Si, Jichao Sun, Kun Lv, Biqing Yan, Bing Li, Xuzheng Zhou, Jiyu Zhang

**Affiliations:** 1 Intensive Care Unit, The Affiliated Hospital of Medical School, Ningbo University, Zhejiang Province Ningbo, People’s Republic of China; 2 School of Medicine, Ningbo University, Ningbo, Zhejiang Province People’s Republic of China; 3 Lanzhou Institute of Husbandry and Pharmaceutical Sciences, Chinese Academy of Agricultural Sciences, Gansu Province Lanzhou, People’s Republic of China; 4 College of Pharmacy, Jinan University, Guangzhou, Guangdong Province People’s Republic of China; 5 College of Veterinary Medicine, Northeast Agricultural University, Harbin, Heilongjiang Province People’s Republic of China; 6 School of Business, Ningbo University, Ningbo, Zhejiang Province People’s Republic of China; 7 Key Laboratory of Veterinary Pharmaceutical Development, Lanzhou Institute of Husbandry and Pharmaceutical Sciences, Chinese Academy of Agricultural Sciences, 730050 Lanzhou, People’s Republic of China

**Keywords:** Myrislignan, Pharmacokinetics, LC-MS/MS, Mouse, Dehydrodiisoeugenol, Bioavailability

## Abstract

**Background:**

Myrislignan is a natural product from Myristica sp. with diverse pharmacological activities. Recently, the anti-*Toxoplasma gondii* (*T. gondii*) activity of myrislignan has been proposed, and *in vivo* studies of its pharmacokinetics in BALB/c mice are necessary to further evaluate the clinical effects of myrislignan.

**Results:**

In this study, a sensitive liquid chromatography-tandem mass spectrometry (LC-MS/MS) method was developed and validated to quantify myrislignan levels in mouse plasma using dehydrodiisoeugenol as an internal standard (IS) in positive ion mode. Chromatographic separation of the analytes was achieved using an ACE Ultracore Super C18 analytical column (2.5 μm, 2.1 × 50 mm) at 30 °C. A gradient mobile phase consisting of water (0.1 % formic acid) and acetonitrile (0.1 % formic acid) was delivered at a flow rate of 0.4 mL/min. Myrislignan and the IS eluted at 1.42 and 1.71 min, respectively. A good excellent linear response across the concentration range of 1-1000 ng/mL was achieved (*r*^2^ = 0.9973). The lower limit of quantification (LLOQ) was 1 ng/mL, and the inter- and intra-day accuracy and precision of the method showed relative standard deviations (RSDs) less than 10 %. The method was applied to examine the pharmacokinetics of myrislignan in mouse plasma following a single oral administration of 200 mg/kg or intraperitoneal administration of 50 mg/kg myrislignan, and the bioavailability (F) of orally administered myrislignan was only 1.97 % of the bioavailability of intraperitoneally administered myrislignan.

**Conclusions:**

A rapid and sensitive LC-MS/MS method has been was developed, validated and successfully used to determine myrislignan levels in mice after oral or intraperitoneal administration. This study is the first to report the pharmacokinetic parameters of myrislignan in mice and to compare its pharmacokinetics after oral and intraperitoneal administration, which will be useful for further research on the administration of myrislignan in animals and humans.

## Background

*Myristica fragrans* Houtt (Myristicaceae) is an aromatic evergreen tree formerly known as a spice [1]. Its seeds, nutmeg, are used not only for cooking but also for medicinal purposes since they have a wide range of pharmacological activities, including anti-inflammatory [2], antibacterial [3], antiangiogenic [4], anticarcinogenic [5], antidiarrhoeal [6] and antiplatelet aggregation [7] activities. Myrislignan is the main active ingredient (greater than 3.5 mg/g crude drug) in nutmeg and was discovered in 1973 [8]. Myrislignan possesses various biological activities since it significantly inhibits nitric oxide production [9]; affects hepatic mixed function oxidase enzyme activity [10]; protects against thioacetamide-induced liver injury [11]; exerts anti-inflammatory [12], antifeeding [8], anticancer [13], and antifungal effects [14]; and inhibits neoplasm formation and vascular smooth muscle contraction [15, 16]. In addition, myrislignan is readily transported in the Caco-2 cell monolayer model [17] and penetrates the blood-brain barrier through passive diffusion [18]. Thus, myrislignan appears to be a promising drug candidate for further pharmaceutical development.

Recently, we identified the anti-*Toxoplasma* activity of myrislignan; thus, *in vivo* studies of its pharmacokinetics are necessary to evaluate the clinical effects of myrislignan. To date, only a few methods for the determination of myrislignan have been reported. One of the pharmacokinetic studies of myrislignan in rats after intravenous administration used high-performance liquid chromatography (HPLC), and the lower limit of quantification (LLOQ) was only 300 ng/mL [19]. In another study, an ultra-high ultrahigh-performance liquid chromatography coupled with mass spectrometry (UPLC–MS) method was developed for the determination of myrislignan levels in rat plasma after oral administration [20]. However, no information about the pharmacokinetics of myrislignan in mice after dosing has been reported. In this study, a simple and sensitive liquid chromatography-tandem mass spectrometry (LC-MS/MS) method was developed for the determination of myrislignan levels in mouse plasma after oral and intraperitoneal administration. The method was successfully used for a comparative pharmacokinetic study of myrislignan after oral administration and intraperitoneal injection in mice, and the bioavailability of myrislignan in mice was calculated.

## Results

### Mass spectrometry and liquid chromatography

**Fig. 1:**
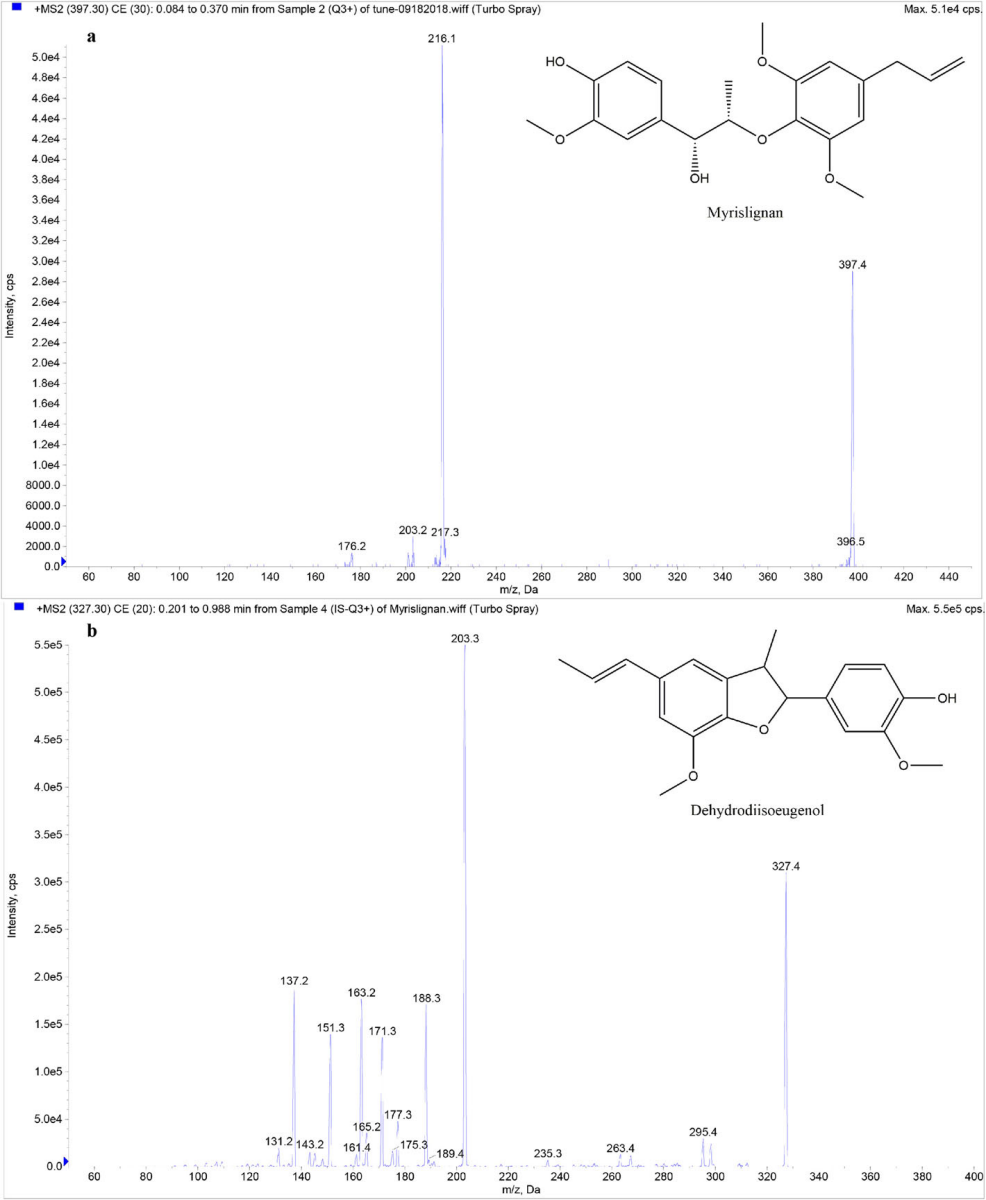
Full-scan product ion spectra of myrislignan (**a**) and dehydrodiisoeugenol (**b**)

To optimize the mass spectrometric parameters of myrislignan and the IS, electrospray ionization (ESI) full scans were performed in both ESI (±) detection modes. Better MS responses were achieved in the ESI (+) mode compared with than in the ESI (-) mode. The scanning MS spectra demonstrated [M + Na]^+^ ions at m/z 397.4 for myrislignan and [M + H]^+^ ions at m/z 327.4 for the IS (Fig. 1). Analyte quantification was performed using the multiple reaction monitoring (MRM) mode to acquire data with high selectivity and sensitivity. In MRM mode, the mass transitions chosen for quantitation were m/z 397.4 to 216.1 for myrislignan and m/z 327.4 to 203.3 for the IS.

Chromatographic separations were conducted on an ACE Ultracore Super C18 analytical column (2.5 μm, 2.1 × 50 mm) at a flow rate of 0.4 mL/min. The analysis was conducted with gradient elution using mobile phase A, 0.1 % formic acid (FA) in water, and mobile phase B, 0.1 % FA in acetonitrile (ACN). Under this these conditions, myrislignan and the IS were well resolved with retention times of 1.42 and 1.71 min, respectively, and endogenous substances in the plasma did not interfere with analyte detection.

### Method validation

#### Selectivity and matrix effects

**Fig. 2:**
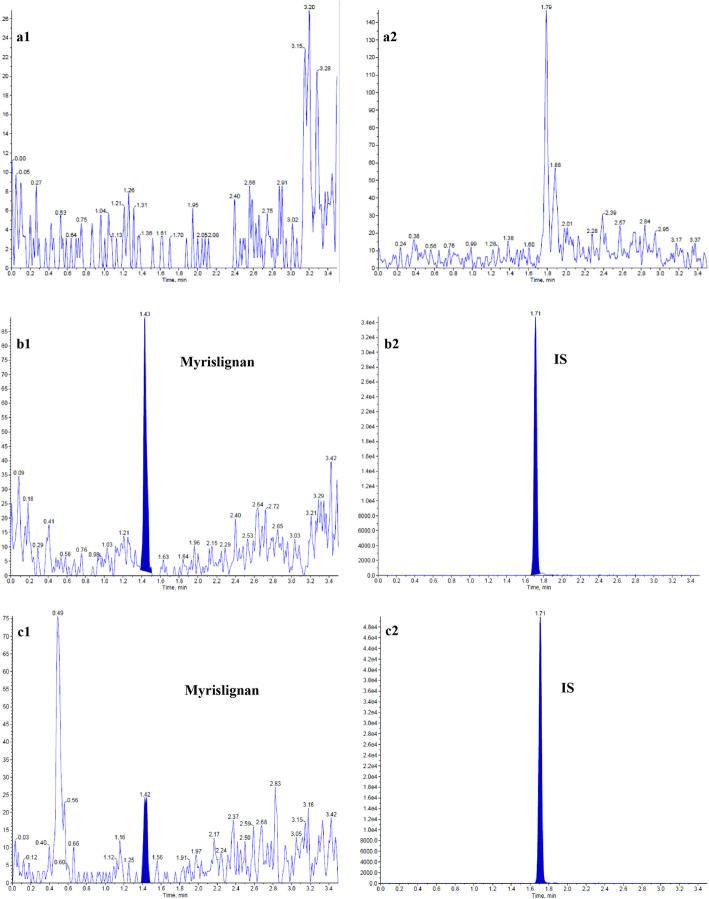
Representative chromatograms of myrislignan and the IS in plasma. **a** blank plasma; **b** a plasma sample obtained after oral administration of 200 mg/kg of myrislignan; **c** a blank plasma sample spiked with the LLOQ

Specificity assays were assessed by analysing blank plasma samples from nine different mice under the above chromatographic conditions. Figure 2 shows representative chromatograms of blank plasma, a real plasma sample collected after oral administration, and a plasma sample at the LLOQ (1 ng/mL). The assay was free of interference from endogenous substance-mediated interference near the retention times of for myrislignan or the IS.

In addition, the mean matrix effect for myrislignan was 91.61 ± 3.63 %, while the matrix effect on the IS was 90.10 ± 2.37 %. The results indicated that there was no significant interference in this assay from the plasma matrix.

#### LLOQ and linearity

**Fig. 3:**
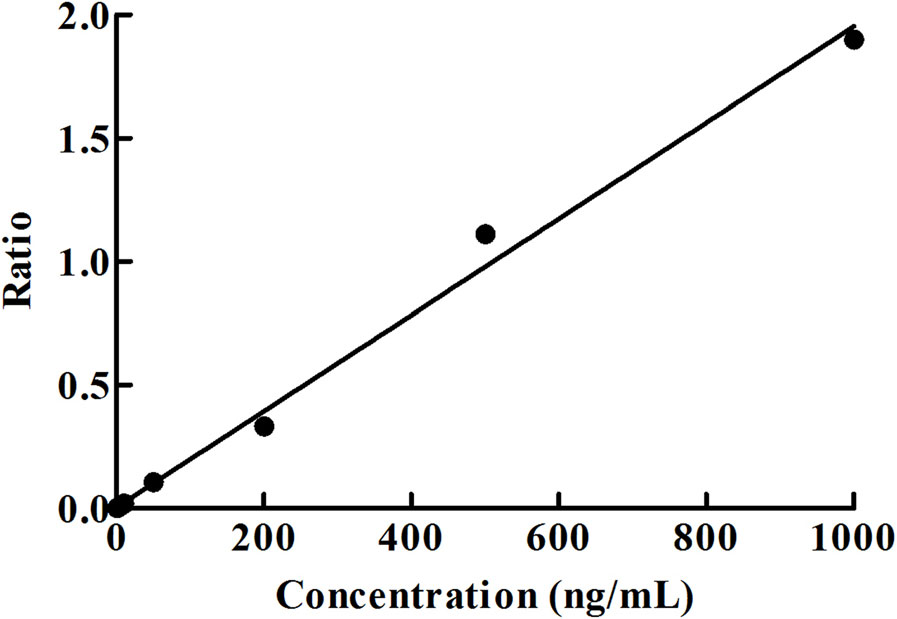
The calibration curves

The LLOQ and lower limit of detection (LLOD) of myrislignan were 1 ng/mL and 0.5 ng/mL, respectively. Furthermore, the calibration curves are were established within the concentration range of 1-1000 ng/mL, as shown in Fig. 3. The typical equation of the calibration curve for myrislignan was y = 0.00202x − 0.00104 (*r*^2^ = 0.9973) with a weighting factor of 1/x^2^, exhibiting an good excellent linearity across the entire calibration range.

#### Accuracy and precision

The intra- and inter‐day precision and accuracy based on the quality control (QC) samples (15 ng/mL, 400 ng/mL and 800 ng/mL) and LLOQ samples (1 ng/mL) are summarized in Table 1. The intraday accuracy for myrislignan ranged from 97.11 to 111.60 %, and the interday accuracy ranged from 99.63 to 109.81 %. The intra- and inter-day precisions were 3.73–7.51 % and 2.72–6.34 %, respectively. The results showed that this method was accurate, reliable and reproducible.

**Table 1:** Intra- and inter-day precision and accuracy of the determination of myrislignan in mouse plasma

Concentration (ng/mL)	Intra-day precision and accuracy (*n* = 6)		Inter-day precision and accuracy (*n* = 18)	
	Accuracy (%) ± SD	RSD (%)	Accuracy (%) ± SD	RSD (%)
1	111.60 ± 7.76	6.96	109.81 ± 4.56	4.15
15	97.11 ± 7.30	7.51	103.57 ± 6.57	6.34
400	108.08 ± 4.02	3.73	99.63 ± 3.04	3.05
800	108.92 ± 5.54	5.08	101.32 ± 2.76	2.72

#### Recovery

The mean extraction recoveries of myrislignan at concentrations of 15.0 ng/mL, 400 ng/mL and 800 ng/mL were 101.83 ± 9.72 %, 101.50 ± 3.06 %, and 109.33 ± 6.79 %, respectively. The mean extraction recovery for IS was 103.76 ± 1.89 %. The results are shown in Table 2, which was demonstrated demonstrating that the extraction method was simple and efficient.

**Table 2:** Recovery of myrislignan (*n* = 6) from mouse plasma

Concentration (ng/mL)		Recovery (%, *n* = 6)	
		Mean (%) ± SD	RSD (%)
Myrislignan	15	101.83 ± 9.72	9.55
	400	101.50 ± 3.06	3.01
	800	109.33 ± 6.79	6.21
IS		103.76 ± 1.89	1.82

#### Stability

The stabilities of myrislignan under different storage conditions are shown in Table 3. The accuracy was 92.22-110.02 %, and the precision (RSD%) was 1.83–7.70 %, indicating that myrislignan was fairly stable under all of the tested conditions.

**Table 3:** Stability of myrislignan in mouse plasma samples under various conditions (*n* = 6)

Storage conditions	Concentration (ng/mL)	Accuracy ± SD (%)	RSD (%)
Ambient temperature for 24 h	15	92.22 ± 6.02	6.53
	400	99.24 ± 4.32	4.35
	800	108.98 ± 3.63	3.33
At -20 °C for 60 days	15	104.21 ± 7.62	7.31
	400	102.35 ± 6.54	6.39
	800	110.02 ± 3.84	3.49
At 4 °C in the autosampler for 24 h	15	106.26 ± 5.43	5.11
	400	108.49 ± 1.98	1.83
	800	99.63 ± 7.67	7.70
3 Freeze-thaw cycles	15	93.02 ± 3.78	4.06
	400	97.85 ± 5.30	5.42
	800	101.73 ± 6.87	6.75

#### Dilution integrity

The plasma concentration of myrislignan after intraperitoneal administration exceeded the linear range. Therefore, the dilution effect on myrislignan was studied. The precision and accuracy of 10-fold diluted plasma samples (*n* = 6) were 6.9 and 106.83 %, respectively.

### Pharmacokinetic studies

**Fig. 4:**
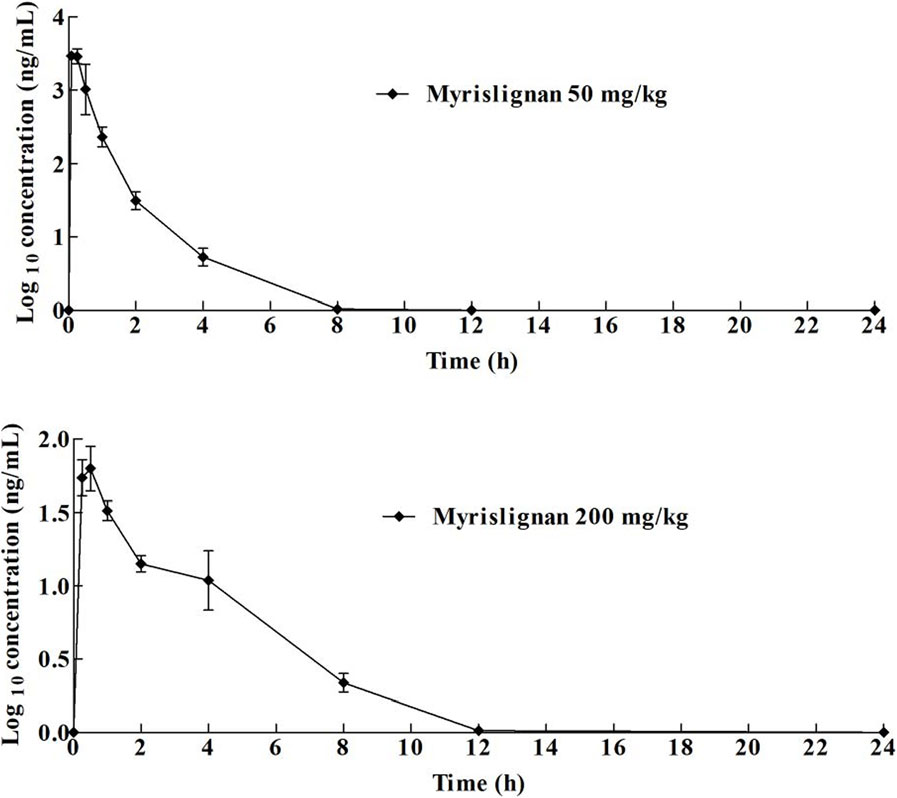
Mean plasma concentration-time profile after administration of myrislignan to mice (*n* = 6)

The plasma concentrations of myrislignan were quantitated after oral or intraperitoneal administration at doses of 200 mg/kg or 50 mg/kg, respectively. The mean plasma concentration–time profiles of myrislignan are shown in Fig. 4. In addition, the major pharmacokinetic parameters of myrislignan after oral or intraperitoneal administration were determined using a non-compartment model and are listed in Table 4. After oral administration of 200 mg/kg myrislignan, the compound reached a half-life After oral administration of 200 mg/kg myrislignan, the compound was absorbed and reached a half‐life (T_1/2_) of 1.97 ± 0.426 h, peak plasma concentration (C_max_) of 105 ± 72.3 ng/mL, peak time (T_max_) of 1.04 ± 1.45 h, and average area under the curve (AUC_0 − t_) of 157 ± 55.1 h/ng/mL. The C_max_ of myrislignan in mice following intraperitoneal administration was 3870 ± 1200 ng/mL, the AUC_0 − t_ of myrislignan in the intraperitoneal group was 2050 ± 532 h/ng/mL, the T_1/2_ was 0.692 ± 0.386 h, and the T_max_ was 0.167 ± 0.0915 h. The bioavailability (F) of orally administered myrislignan was only 1.97 % compared with that of the intraperitoneal group.

**Table 4:** The pharmacokinetic parameters of myrislignan in mice following oral or intraperitoneal administration (*n* = 6)

Parameters (Units)	Mean ± SD	
	Intraperitoneal	Oral
K_el_ (h^− 1^)	1.19 ± 0.443	0.366 ± 0.0789
T_1/2_ (h)	0.692 ± 0.386	1.97 ± 0.426
T_max_ (h)	0.167 ± 0.0915	1.04 ± 1.45
C_max_ (ng/mL)	3870 ± 1200	105 ± 72.3
Cl_F_obs_(mL/hr/kg)	45,123 ± 11,936	302,900 ± 442,668
Vz_F_obs_(mL/kg)	24,198 ± 5987	970,149 ± 199,518
AUC_0 − t_ (h/ng/mL)	2050 ± 532	157 ± 55.1
AUC_0 − inf_ (h/ng/mL)	2060 ± 536	162 ± 52.9
AUMC_last_ (hr*hr*ng/mL)	1006 ± 227	457 ± 240
MRT_0 − t_ (h)	0.457 ± 0.0499	2.19 ± 0.841
MRT_0 − inf_ (h)	0.466 ± 0.0485	2.57 ± 0.858
F* = 1.97 %		

## Discussion

In the method development stage, because the detection limit of HPLC is too high to accurately evaluate the pharmacokinetics level of myrislignan in mice, the LC-MS/MS method was used to meet the experimental requirements. Under the optimal LC conditions, FA was added to increase the ionization of all of the tested compounds and to improve the peak shape. The composition of the mobile phase in this study was deemed suitable for the separation and ionization of myrislignan and the IS, as since it provided a good peak shape and resolution. In addition, procedures used to validate the optimized bioanalytical method for myrislignan were performed in accordance with FDA guidelines [21]. The LC-MS/MS method was validated in terms of selectivity, linearity, matrix effect, LLOQ, recovery, accuracy and precision, stability and dilution integrity. Here, a good well linear relationship between the ratio of the myrislignan concentration and the quantitative ion peak area was established. The method displayed satisfactory accuracy, precision and reproducibility for the quantification of myrislignan levels in mouse plasma, and no endogenous substance-mediated interference was observed. Liquid-liquid extraction with acetonitrile was both simple and efficient, and the plasma samples containing concentrations greater than the maximum value of the calibration curve were also accurately measured. In addition, myrislignan was stable in mouse plasma after exposure to different storage conditions. Thus, the LC-MS/MS method developed and validated in our study was simple and sensitive.

The pharmacokinetic parameters of myrislignan in mouse plasma were successfully obtained in this study. Based on previously published data obtained after the oral administration of 18.3 mg/kg myrislignan [22], the C_max_ and AUC_0 − t_ in rats are were 102.44 ± 16.51 ng/mL and 158.09 ± 40.40 h/ng/mL, respectively, while the pharmacokinetic properties obtained for myrislignan after oral administration at a dose of 200 mg/kg in mice were C_max_ and AUC_0 − t_ values of 105 ± 72.3 ng/mL and 157 ± 55.1 h/ng/mL, respectively. The results obtained after oral administration in mice were all lower than those obtained after intraperitoneal administration (50 mg/kg), showing C_max_ of 3870 ± 1200 ng/mL and AUC_0 − t_ of 2050 ± 532 h/ng/mL. These results indicated that the oral absorption of myrislignan was limited. However, a higher MRT_0 − t_ value was observed in the oral administration group than in the intraperitoneal group. These results highlight emphasize that the pharmacokinetics of myrislignan in mice were characterized by rapid absorption and metabolism, as well as poor oral absorption since its oral bioavailability was only 1.97 %, and they suggest that intraperitoneal injection could achieve a higher blood concentration. Recently, we observed the anti-*T. gondii* activity of myrislignan. The pharmacokinetic data presented in the present study could provide a basis for further clinical studies of the pharmacodynamics of myrislignan.

## Conclusions

A rapid and sensitive LC-MS/MS method has been developed, validated and successfully used to determine myrislignan levels in mice after oral or intraperitoneal administration. The sample preparation procedure for the assay is simple and relatively quick, and the analysis method provides excellent sensitivity, linearity, precision and accuracy. This method was successfully used in the pharmacokinetic evaluation of myrislignan in mice after oral or intraperitoneal administration. This study is the first to report the pharmacokinetic parameters of myrislignan in mice and to compare its pharmacokinetics after oral and intraperitoneal administration, which could be useful for further research on the administration of myrislignan in animals and humans.

## Methods

### Reagents

Standard myrislignan and dehydrodiisoeugenol were provided by Desite Biotechnology Co., Ltd. (Chengdu, China) (batch numbers DST180502-043 and DST180310-055, respectively, purities > 98 %). Chromatographic grade ACN and FA were purchased from Fisher Chemical (Waltham, MA, USA). Water was purified with a Milli-Q Plus water system (Millipore Corporation, Bedford, MA, USA) before use. All of the other chemicals and reagents were of chromatographic grade.

### Instrumentation

The LC-MS/MS instrument (API 4000) consisted of an LC system with a binary pump (Model SL) and a triple-quadrupole mass spectrometer with an ESI interface (Agilent Technologies Inc., Santa Clara, CA, USA). The system was controlled using Mass Hunter Analyst software (version 1.6.2, Agilent Technologies Inc.).

### LC/MS/MS analysis

The analytes were separated on an ACE Ultracore Super C18 column (2.1 × 50 mm, 2.5 μm) (ACE Chromatography Technology Co., Ltd., UK); mobile phase A (0.1 % FA in water) and mobile phase B (0.1 % FA in ACN) were used. The mobile phase was delivered at a flow rate of 0.4 mL/min, and the following gradient profile was used: (1) 0–0.50 min, 45 % B; (2) 1.30–2.30 min, 95 % B; (3) 2.31 min, 45 % B; and (4) 3.50 min, stop. The sample was injected at a volume of 10 µL at 30 °C.

The Mass spectrometry analysis was performed in positive ion mode. The following MS/MS parameters were used: ion spray voltage, 5 kV; source temperature, 500 °C; curtain gas pressure, 20 psi; nebulizer gas (GS1) pressure, 55 psi; and auxiliary nitrogen gas (GS2) pressure, 55 psi. The declustering potentials (DPs) for myrislignan and IS were 64 and 70 V, respectively. Data were collected in MRM mode using [M + Na]^+^ ions for myrislignan and [M + H]^+^ ions for the IS, with collision energies (CE values) for the transitions of m/z 397.4→216.1 and m/z 327.4→203.3 of 30 eV and 19 eV, respectively. The entrance potential (EP) for both myrislignan and IS was 10 V, and the collision cell exit potentials (CXP) for myrislignan and the IS were 12 and 13 V, respectively.

### Preparation of working, calibration and quality control standard solutions

For the standard stock solution of myrislignan, 100 mg of myrislignan were placed into a 100 mL brown volumetric flask, after which methanol was added to produce a stock solution of 1000 µg/mL myrislignan. Standard working solutions were prepared from the myrislignan stock solution by dilution with methanol and water (4:1). Calibration standards were prepared by diluting the corresponding standard working solutions with blank mouse plasma to achieve final concentrations of 1.00, 2.00, 5.00, 10.0, 50.0, 200, 500 and 1000 ng/mL. QC samples with concentrations of 1 ng/mL (LLOQ), 15 ng/mL (LQC, low-quality control), 400 ng/mL (MQC, medium-quality control) and 800 ng/mL (HQC, high-quality control) were prepared in a similar manner.

For the IS solution, 100 mg of IS were placed into a 100 mL brown volumetric flask, and methanol was added to produce a stock solution of 1000 µg/mL IS. The IS stock solution was further diluted with acetonitrile to prepare the working solution (50 ng/mL). All of the solutions were stored at 4 °C and brought to room temperature before use [23].

### Sample preparation

A 20 µL aliquot of sample was added to 180 µL of ACN containing the IS (IS, 50 ng/mL), and the mixture was vortexed for 10 min and then centrifuged at 3700 rpm for 10 min. Then, 80 µL of the supernatant were added to 80 µL of water and vortexed for 10 min, after which the solution was filtered through a 0.22 μm Millipore filter. A 10 µL aliquot of the mixture was injected into the LC-MS/MS system.

### Method validation

The LC-MS/MS method was validated in terms of the selectivity, linearity, matrix effect, LLOQ, recovery, accuracy, precision, stability and dilution integrity.

#### Selectivity and matrix effects

Selectivity was evaluated by analysing nine different batches from multiple types of mice to confirm the absence of interfering peaks. The presence of carryover was tested by subjecting a blank sample to chromatographic separation after a myrislignan sample had been analysed to determine whether any similar peaks appeared in the chromatogram of the blank sample [24].

The matrix effect was defined as the response ratios of analyte dissolved in pretreated blank matrix samples spiked with myrislignan at three QC levels with standard solutions of equivalent concentrations that had been directly dried and reconstituted with the same mobile phase [25].

#### LLOQ and linearity

The LLOQ and the LLOD were calculated based on the ratios of signal to baseline noise (S/N) of least 10 and 3, respectively. The calibration curve for myrislignan was linear at concentrations ranging from 1.000 to 1000 ng/mL, and a coefficient of correlation (r^2^) of at least 0.99 was required to meet the criterion. The calibration curve was constructed by plotting the ratio of the peak areas of myrislignan/IS (y) against the nominal concentration of myrislignan (x) in the form of y = ax + b, and the least squares method was used for the linear regression analysis [23].

#### Accuracy and precision

The precision method was determined as the RSD of replicate measurements, and accuracy was evaluated as the ratio of the calculated and theoretical concentrations, as previously described [25]. The intraday accuracy and precision of the HPLC-MS/MS method were assessed by analysing QC samples at concentrations of 15.0 ng/mL, 400 ng/mL and 800 ng/mL, and the LLOQ concentration was assessed using six replicates per concentration on the same day. Interday accuracy and precision were evaluated by analysing QC solutions at concentrations of 15.0 ng/mL, 400 ng/mL, and 800 ng/mL and the LLOQ concentration with six determinations per concentration over 3 days [26]. According to the ICH [27], the criterion for precision and accuracy was that the RSD should be ≤ 15 % for each concentration except the LLOQ (≤ 20 %).

#### Recovery and stability

The recovery was determined by comparing the analytical results of the extracted QC samples with the pure standards without extraction. Three concentrations of QC samples (15.0 ng/mL, 400 ng/mL and 800 ng/mL) were evaluated with six replicates per concentration to evaluate the efficiency of myrislignan extraction from the biological matrix.

The stability of myrislignan exposed to mouse plasma was investigated by analysing replicates (*n* = 6) of the QC samples at concentrations of 15.0 ng/mL, 400 ng/mL and 800 ng/mL under various sample storage and processing conditions [28]: (1) kept at 25℃ for 4 h (ambient stability); (2) stored at -20℃ for 60 days (long-term stability); (3) kept in the autosampler for 24 h at 4℃ (autosampler stability); and (4) three freeze/thaw cycles (-20℃ to 25℃, freeze-thaw stability) [26].

#### Dilution integrity

The dilution integrity of myrislignan in plasma was studied to determine whether myrislignan was accurately quantified at concentrations exceeding the maximum value of the calibration curve. Plasma samples (*n* = 6) at concentrations ranging from 4000 to 1000 ng/mL were diluted ten-fold with blank plasma. The diluted samples were further quantified with respect to the calibration curve. The accuracy and precision of the determination after dilution should be within the acceptable limit (RSD%, 15 %).

### Pharmacokinetic study in mice

Thirty-six healthy BALB/c mice of both sexes with body weights (BW values) of 18.00 ± 2.00 g were purchased from Beijing Vital River Laboratory Animal Technology Co., Ltd. (animal certification number: male, 11400700331244; female, 11400700331308), and acclimatized to a standard, environmentally controlled animal room (temperature, 25 ± 2 °C; relative humidity of 50 % and a 12:12-h light/dark cycle) for 1 week before the experiment. Adequate water and food were provided. All of the experimental methods, the animal care and the barn environment of this study strictly complied with the Guide for the Care and Use of Laboratory Animals, Lanzhou Institute of Husbandry and Pharmaceutical Sciences, China. The experiment has been approved by the institutional ethics committee of Lanzhou Institute of Husbandry and Pharmaceutical Sciences, China, and the certificate number was SCXK (Gan) 2019-0010. In addition, all efforts were made to minimize suffering. At the end of the experiment, mice were moved into a carbon dioxide death chamber, anesthetised with sevoflurane, and then humanely killed with carbon dioxide. During the operation, the mice were as gentle as possible to minimize the suffering. The mice were confirmed to be motionless and not breathing, and were confirmed to be dead after two minutes of observation.

Animals were administered myrislignan once, and the dose was based on the live weight. For intraperitoneal injections, myrislignan was dissolved in isotonic saline containing 10 % ethanol and 10 % Cremophor EL at a concentration of 5 mg/mL. For oral administration, myrislignan was suspended in 0.5 % carboxymethyl cellulose and 0.5 % Tween 80 to a final concentration of 20 mg/mL. Mice were randomly divided into two groups (*n* = 18 animals per group). One group received an oral dose of 200 mg/kg myrislignan, and blood samples (80 µL) were collected int heparinized centrifuge tubes from the orbital sinus venous plexus at 0, 0.25, 0.5, 1, 2, 4, 8, 12 and 24 h post-dose. Mice in the other group received an intraperitoneal injection of 50 mg/kg myrislignan, and blood samples (80 µL) were collected in heparinized centrifuge tubes from the orbital sinus venous plexus at 0, 0.083, 0.25, 0.5, 1, 2, 4, 8, 12 and 24 h post-dose postdose. Each blood sample was centrifuged at 3000 rpm for 10 min, and then a plasma sample was collected and immediately stored in a − 20 °C freezer until analysis using LC-MS/MS.

The best pharmacokinetic model was confirmed according to the minimum Akaike Information Criterion (AIC) value principle and utilized for data fitting and parameter estimation [29]. Accordingly, the pharmacokinetic parameters were estimated by a noncompartmental model using WinNonlin Professional software, version 8.0 (Pharsight, Mountain View, CA, USA). The K_el_, C_max_, T_1/2_, T_max_, MRT_0 − t,_ MRT_0 − inf_, and area under the concentration–time curve from time zero to the last time point (AUC_0 − t_) and to infinity (AUC_0 − inf_) were calculated from the data. The oral bioavailability, F (%), of myrislignan was evaluated using the following formula [30]:

1 $$ \mathrm{F}\left(\%\right)=\frac{\mathrm{AUC}\left(\mathrm{p}.\mathrm{o}.\right)/\mathrm{Dose}\left(\mathrm{p}.\mathrm{o}.\right)}{\mathrm{AUC}\left(\mathrm{i}.\mathrm{p}.\right)/\mathrm{Dose}\left(\mathrm{i}.\mathrm{p}.\right)}\times 100\% $$

where AUC_(p.o.)_ and AUC_(i.p.)_ represent the values for AUC_(0−inf)_ in the oral and intraperitoneal administration groups, respectively.

The experiment has been approved by the institutional ethics committee of Lanzhou Institute of Husbandry and Pharmaceutical Sciences, China, and the certificate number was SCXK (Gan) 2019-0010. All of the experimental methods, the animal care and the barn environment of this study strictly complied with the Guide for the Care and Use of Laboratory Animals, Lanzhou Institute of Husbandry and Pharmaceutical Sciences, China. The study was carried out in compliance with the ARRIVE guidelines.

## Data Availability

All of the data generated or analysed during this study are available from the corresponding author on reasonable request.

## Abbreviations

AUC_0 − t_: Area under the concentration-time curve from time zero to the last time point
AUC_0 − inf_: Area under the concentration-time curve from time zero to infinity
BW: Body weight
C_max_: Peak plasma concentration
ESI: Electrospray ionization
HPLC-MS/MS: High-performance liquid chromatography-tandem mass spectrometry
IS: Internal standard
LC-MS/MS: Liquid chromatography coupled with tandem mass spectrometry
LLOD: Lower limit of detection
LLOQ: Lower limit of quantification
MRM: Multiple reaction monitoring
QC: Quality control
RSD: Relative standard deviation
Tmax: Peak time
T_1/2_: Half-life
F: Bioavailability
HPLC: High-performance liquid chromatography
FA: Formic acid
ACN: Acetonitrile
DP: Declustering potentials
EP: Entrance potential
CXP: Collision cell exit potentials
CE: Collision energy
LQC: Low quality control
MQC: Medium quality control
HQC: High quality control
r^2^: Coefficient of correlation
